# Physical Chemistry Study of Collagen-Based Multilayer Films

**DOI:** 10.3390/gels9030192

**Published:** 2023-03-02

**Authors:** Yi-Wei Chen, Muhammad Haseeb Iqbal, Florent Meyer, Vincent Ball, Fouzia Boulmedais

**Affiliations:** 1 Institut Charles Sadron (UPR 22), Université de Strasbourg, CNRS, 67034 Strasbourg, France; 2 Centre de Recherche en Biomédecine de Strasbourg, Institut National de la Santé et de la Recherche Médicale, 67085 Strasbourg, France; 3 Faculté de Chirurgie Dentaire, Université de Strasbourg, 67000 Strasbourg, France

**Keywords:** collagen, polyphenols, layer-by-layer assembly, polyelectrolyte, biocompatible

## Abstract

The surface properties of a biomaterial play an important role in cell behavior, e.g., recolonization, proliferation, and migration. Collagen is known to favor wound healing. In this study, collagen (COL)-based layer-by-layer (LbL) films were built using different macromolecules as a partner, i.e., tannic acid (TA), a natural polyphenol known to establish hydrogen bonds with protein, heparin (HEP), an anionic polysaccharide, and poly(sodium 4-styrene sulfonate) (PSS), an anionic synthetic polyelectrolyte. To cover the whole surface of the substrate with a minimal number of deposition steps, several parameters of the film buildup were optimized, such as the pH value of the solutions, the dipping time, and the salt (sodium chloride) concentration. The morphology of the films was characterized by atomic force microscopy. Built at an acidic pH, the stability of COL-based LbL films was studied when in contact with a physiological medium as well as the TA release from COL/TA films. In contrast to COL/PSS and COL/HEP LbL films, COL/TA films showed a good proliferation of human fibroblasts. These results validate the choice of TA and COL as components of LbL films for biomedical coatings.

## 1. Introduction

The surface properties of a biomaterial play an important role in cell behavior, e.g., recolonization, proliferation, and migration. They can be modified in several ways by playing on the fabrication process such as by introducing bioactive molecules onto the surface, by chemical grafting, adsorption, and electrospinning methods [1,2]. Amongst these surface modification techniques, layer-by-layer (LbL) is a versatile way to fabricate and functionalize surfaces by the consecutive deposition of oppositely charged molecules through electrostatic interaction by either dip coating or spray coating [3]. It is a powerful tool that allows the fabrication of thin films with precise control over properties such as thickness, roughness, surface chemistry, and biocompatibility. They can be designed by depositing a variety of different biocompatible polyelectrolytes, such as chitosan, alginate, chondroitin sulfate, poly(L-lysine), and collagen (COL), onto different substrates [4]. Exponentially growing LbL films behave as 2D gels, as it was reported for polysaccharide [5] or collagen-based films [6,7]. In particular, they have a buildup transition from droplets to a smooth homogenous film [5] with the increase in number of deposition steps and a Young’s modulus of a few kPa [8,9]. LbL films are fabricated at room temperature using aqueous solutions, allowing the incorporation of a wide range of proteins and functional biopolymers without changing their bioactivity [10].

Numerous applications, such as drug delivery, tissue regeneration, and viscosupplementation, have been developed within the concept of LbL techniques [4,11]. LbL films can have the same role as the extracellular matrix in promoting the proliferation of epithelial cells, for example, on the wound site, and can tackle bacterial infection using antibacterial polymers. COL, polycationic and soluble at acidic pH values, is the most abundant protein in mammals and the main component of the extracellular matrix in the skin. It is a biocompatible, biodegradable, and nontoxic polymer. Several studies have shown that COL could induce wound healing by promoting the recolonization and the proliferation of epithelial cells with good hemostatic, low antigenicity, and low inflammatory properties [12,13,14]. However, tissue matrix metalloproteases and bacterial infection may result in the enhancement of the breakdown of COL-based material [14,15]. COL-based fibrillar nano-microstructures may mimic basement membranes *in vivo*, where the fibrillar structures similar to COL are important [16]. Built in an acidic pH due to COL solubility issues, COL-based LbL films were developed with anionic polyelectrolytes, such as alginate [16,17], heparin [18,19,20], chondroitin sulfate [19,21], hyaluronic acid [22,23,24], and poly(styrene sulfonate) [25] or with hydroxyapatite [26]. Unstable under physiological conditions, these films require a cross-linking step using carbodiimide chemistry [27], glutaraldehyde, or genipin [16]. Cross-linking agents could compromise the cellular functions of collagen [16]. Therefore, it is important to develop robust COL-based materials. Tannic acid (TA), a natural polyphenol, is a natural molecule extracted from plants, in particular the galls of Rhus and Quercus species [14]. It has antibacterial, antimicrobial, antiviral, as well as anti-inflammatory properties. In addition, it can complex with or crosslink proteins, further becoming a protective layer of the epithelial tissue [28,29]. Recently, we reported a buffer-dependent antibacterial property of COL/TA films. Based on hydrogen bonds between TA and COL, the films were stable in physiological conditions and have a release-killing effect towards *Staphylococcus aureus* thanks to the local TA release when the films were built using citrate buffer [30].

The present work focuses on the optimization of COL/TA film buildup parameters to obtain a homogeneous film covering the entire substrate with a limited number of deposition steps. In comparison, electrostatic self-assembled COL/poly(sodium 4-styrene sulfonate) (PSS), and COL/heparin (HEP) LbL films were also studied. The dipping time, the pH of the buffer, and sodium chloride (NaCl) concentration were varied. Built at an acidic pH, the stability of the films was studied in physiological conditions, as well the release of TA. The morphology of the films was characterized by atomic force microscopy (AFM). Finally, an analysis of the cytotoxicity towards human fibroblasts was performed by comparing the different COL-based films.

## 2. Results and Discussion

### 2.1. Optimization of COL/TA Film Buildup

**Figure 1:**
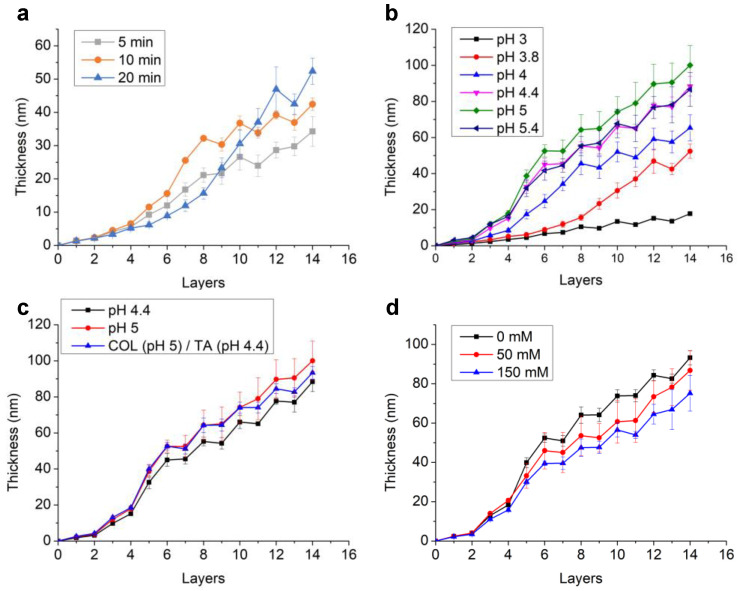
Buildup of COL/TA LbL films: evolution of (COL/TA)_7_ film thickness measured by ellipsometry (**a**) at pH 3.8 with different dipping times, (**b**,**c**) at different pH values with a dipping time of 20 min, and (**d**) with COL at pH 5 and TA at pH 4.4 at different NaCl concentrations with a dipping time of 20 min. The deposition of TA and COL corresponds to even and odd numbers, respectively. Mean and standard deviation values were determined from the thickness of three different samples, with this thickness being the average of 10 values from 10 different areas of the sample.

**Effect of the deposition time**. The optimization of COL/TA film buildup was performed by varying several parameters and following the film thickness by ellipsometry (Figure 1). Different adsorption times of COL and TA were investigated at pH 3.8 in citrate buffer (Figure 1a). Two growth regimes were observed. The first regime was exponential growth over the first 8 steps for 5 min and 10 min of dipping and up to 14 steps for 20 min of dipping. In this regime, COL contributed more to the film thickness than TA, in particular with 10 min of dipping. With 20 min of dipping, the increase in the thickness of both COL and TA was small, which led to the lowest accumulation. The contact with COL (or TA) solutions led to the formation of COL/TA complexes on the surface of the film [30]. The strong interaction between COL and TA likely removed the complexes from the surface due to a stronger interaction of the complexes with the macromolecules present in solution than with the surface of the film. Afterwards, in the case of 5 and 10 min of dipping, the growth became linear with the film thickness, increasing with each dipping in TA and decreasing with each dipping in COL. Such an exponential-to-linear transition was reported for electrostatic-based LbL assembly, such as hyaluronic acid/poly(l-lysine) [31], and hydrogen-bonded LbL films, such as poly(acrylic acid)/poly(vinylpyrrolidone) (PAA/PVPON) [32] and PAA/poly(2-ethyl-2-oxazoline) [33]. This was explained by a film restructuration that prohibited the diffusion of one of the polyelectrolytes. After 14 layers, the highest film thickness was observed for 20 min dipping time. Hence, an adsorption time of 20 min was selected in the following experiment.

**pH influences the buildup of COL/TA films**. Different pH values of citrate buffers were further used to dissolve COL and TA. Due to solubility issues of COL, the pH was set at a maximum of 5.4. At an adsorption time of 20 min, the growth rate of COL/TA LbL films increased with the pH of the solutions reaching an optimum at pH 5 (Figure 1b). This might have been due to the self-assembly property of collagen in solution when the pH was near its isoelectric point (pI = 5.5) [34]. It has been reported that larger-diameter self-assembled collagen fibers could be formed in higher pH environments [35] and citrate buffer [36]. At pH 5.4, the rate of COL/TA film buildup was similar to that at pH 4.4 but lower than that at pH 5. The poor solubility of COL near the pI (pH 5.4) might have resulted in the lower concentration of protein in the solution, leading to a smaller deposition of COL. It has to be noticed that COL solutions were filtered before use to remove undissolved COL aggregates. The transition from exponential to linear could be seen with the linear growth starting from the eighth step at pH 4 and the sixth step for the other pH values (Figure 1b).

In the linear regime, the thickness increment in TA was higher at pH 4.4 than at pH 5. This could be explained by the fact that hydrogen bonds are favored at pHs below the pKa of TA (pKa = 6). Indeed, in the case of hydrogen-bonded LbL, an increase in the ionization state of the polyanion leads to an increase in film thickness with pH [37]. Regarding these results, COL/TA LbL buildup was studied with COL solution prepared at pH 5 and TA at pH 4.4 (Figure 1c). The film reached a thickness of 90 nm after seven pairs of layers with better reproducibility in comparison to the buildups performed at pH 5 and pH 4.4. We thus fixed the pH of COL solution at 5 and TA solution at 4.4.

**The ionic strength decreases COL/TA film thickness.** It is well known that the ionic strength of the solution may influence the growth rate of the LbL film [38]. In the case of COL/TA films, the film thickness decreases with the increase in NaCl concentration due to a lower deposition of both TA and COL (Figure 1d). The poor solubility of COL in the presence of NaCl leading to a lower concentration of COL in solution could be the reason for this. Regarding TA, it was reported for PVPON/PAA H-bonded LbL films that the addition of sodium chloride led to the reduced hydration of the PAA polymer chains and thus reduced deposition [39]. Therefore, in the following experiment, COL and TA solutions were prepared in pure water at pH 5 and pH 4.4, respectively.

### 2.2. Surface Morphology and Effect of the Drying Step on COL/TA Film Thickness

**Figure 2:**
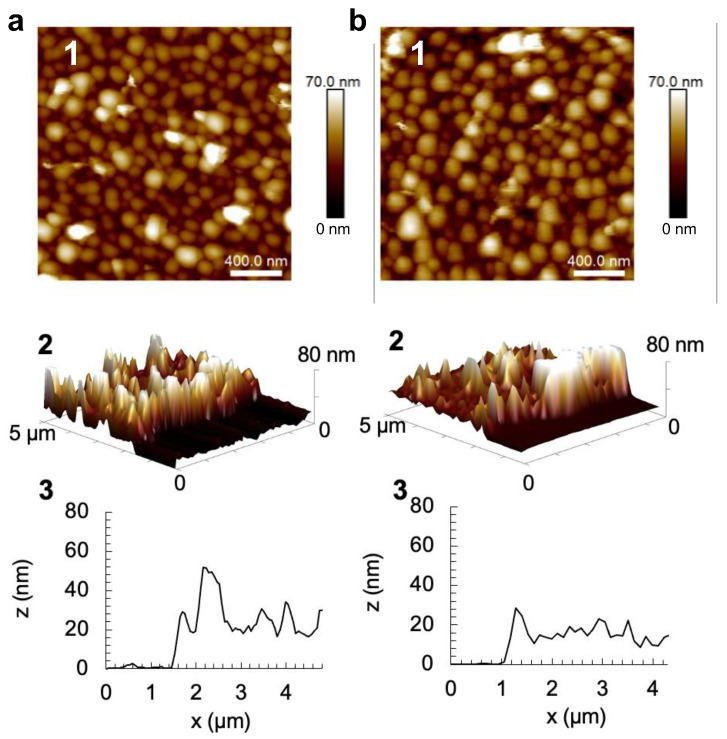
AFM characterization, in contact mode and the liquid state, of (**a**) (COL/TA)_3_ and (**b**) (COL/TA)_3_-COL films: (1) surface topography, (2) AFM 3D images, and (3) cross-section profiles of scratched films in their respective pH buffer.

The surface morphology of COL/TA films was characterized by AFM in the liquid state when ended by TA and COL with no significant difference from both films (Figure 2). Instead of COL fibers, small round aggregates were observed, which were due to the strong complexation between COL and TA through hydrogen bonds and hydrophobic interactions [30]. No significant difference in the granular topography of the multilayers with an increasing number of bilayers was observed [30]. The films were built using two different protocols: dried COL/TA films were obtained by applying a drying step after each rinsing step, and non-dried COL/TA films were obtained without applying the drying step. Scratching of the films allowed us to determine their thickness by AFM. Observed in a cross-section profile measured within one or two areas of one sample, the wet thicknesses of non-dried (COL/TA)_3_ and (COL/TA)_3_-COL films were between 24 and 36 nm, and the dried films were around 60 nm (Table 1). Heterogeneous dried COL/TA films were observed due to the thicker part and thinner parts of the films generated during the buildup. The ellipsometry measurements showed that the dry thickness of the non-dried (COL/TA)_3_ and (COL/TA)_3_-COL films was significantly lower than the dried ones (Table 1). The drying process through compressed air has an influence, as shown in several studies, on the structure [40,41], degree of ionization [42], roughness [43], and thickness [41,44] of LbL films. Although non-dried films have a smaller thickness than dried ones, they are more reproducible and homogeneous. After three bilayers, non-dried COL/TA films could be used for cell cytotoxicity tests as the substrate was fully covered, as confirmed by the AFM cross-section profile (Figure 2).

### 2.3. Stability of COL/TA Film and TA Release in Physiological Medium

**Figure 3:**
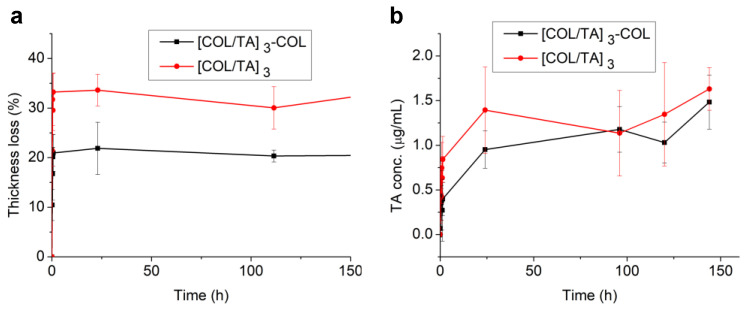
Stability of COL/TA films and TA release. (**a**) Thickness loss of non-dried films on silicon measured by ellipsometry in the dry state and (**b**) concentration of TA released from non-dried films deposited on glass slides and put in contact with a physiological medium.

COL-based LbL films built at an acidic pH, such as the (COL/ hyaluronic acid) films, are not stable when the pH is raised to 7.4 due to the deprotonation of collagen [22]. Before the cytotoxicity tests, the stability of COL/TA films and the release of TA were evaluated in physiological conditions (dipping in a buffer solution at pH 7.4 and 0.15 M NaCl). The thickness of (COL/TA)_3_ and (COL/TA)_3_-COL films decreased by about 33% and 20% of the original thickness, respectively, reaching a plateau after 30 min (Figure 3a). Almost the same quantity of TA (1.7 µg/mL, 1 µM) was released from both films after 150 h of contact (Figure 3b). In 20 h, more TA was released from (COL/TA)_3_, probably from the outer layer, leading to a higher kinetic of release compared to (COL/TA)_3_-COL.

### 2.4. Buildup and Stability Tests of COL/PSS and COL/HEP Films

Two other COL-based films were built using PSS, an anionic synthetic polyelectrolyte, and HEP, a natural anionic polysaccharide with anticoagulant properties derived from mammal mucosal tissues. At a deposition time of 20 min, the pH of the solutions and the salt concentration were varied. The highest growth rate of COL/PSS film was obtained at pH 5 in pure water (Figure S1a in the supporting information, SI). In the case of COL/HEP film, the most reproducible buildup in pure water was obtained at pH 3 (Figure S1b in the SI). The presence of NaCl induced an increase in the film thickness. At pH 3 and 150 mM NaCl, the films had the highest growth rate but were heterogeneous. Thus, we chose to build COL/PSS films at pH 5 in pure water and COL/HEP films at pH 3 and 50 mM NaCl. The topography of the COL/PSS and COL/HEP films was fibrillar and granular, respectively (Figure S2 in SI). The thicknesses of both films measured by AFM and ellipsometry are shown in Table S1 in the SI. In the cross-section of AFM images of scratched films, fully covered substrates were observed for three and seven pairs of layers, respectively (Figure S2 in SI). Dried COL/PSS and COL/HEP films were put in contact with a physiological solution to test their stability (Figure S3 in the SI). COL/PSS film was the most stable film compared to COL/HEP and COL/TA.

### 2.5. In Vitro Cytotoxicity—Effect of the Chemical Composition of COL Containing Multilayers

**Figure 4:**
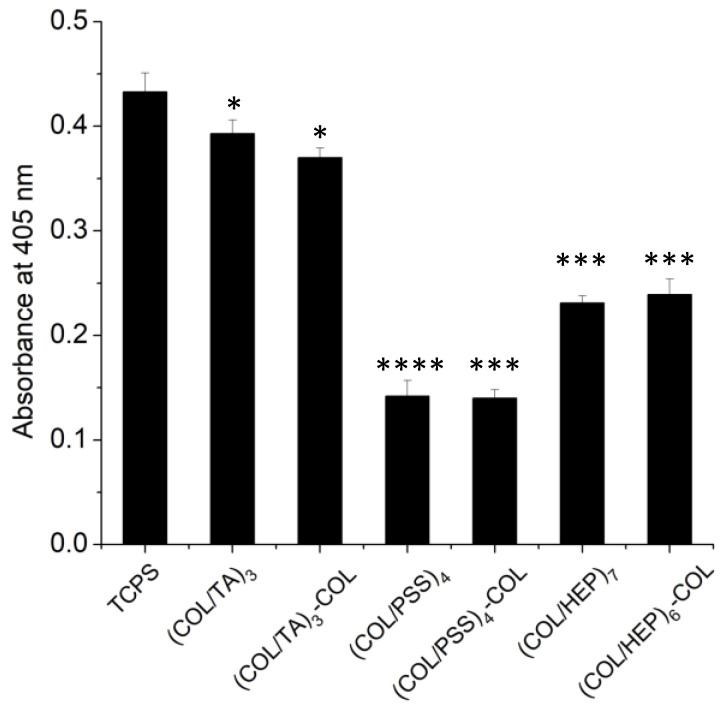
Cytotoxicity of the COL-based films towards human fibroblast cells. Acidic phosphatase assay of cells incubated for 22 h on different COL-based films and tissue culture polystyrene. The data represent the mean and standard deviation of the experiments conducted in triplicate. The two-tail Student’s *t*-test was calculated on the absorbance values of individual experiments in comparison to the TCPS (* *p* ≤ 0.05, *** *p* ≤ 0.001, and **** *p* ≤ 0.0001).

To evaluate the cytotoxicity of the COL-based films, human fibroblast cells (BJ cell line, ATCC CRL-2522) were seeded on COL/TA, COL/PSS and COL/HEP for 22 h. Their mitochondrial activity was evaluated by absorbance using the acidic phosphatase assay (Figure 4) with tissue culture polystyrene (TCPS) used as a control. The cellular activity was slightly lower on COL/TA film compared to the control, representing 91 and 86% of the absorbance value of TCPS. COL/TA films showed no cytotoxicity independently of the ending layer. Noteworthily, the concentration of TA released (1 µM) from these films was less than its reported cytotoxic concentration (125 µM) towards NIH3T3 mouse fibroblasts [45]. At low concentrations, TA was reported to improve cell adhesion [46] and differentiation [36]. The release of more TA in the first 20 h led to better activity on TA-ended films than COL-ended ones.

COL/PSS (0.140 ± 0.011) and COL/HEP (0.235 ± 0.011) films led to significantly lower cell activity compared to TCPS, representing 32% and 54% of the control absorbance value, respectively. COL/PSS and COL/HEP show, to some extent, cytotoxicity toward human fibroblasts. One could hypothesize that the cell adhesion is impeded by the masking of collagen cell binding sites by PSS or HEP [47]. However, the results are similar whatever the ending layer, which invalidates this hypothesis and confirms the cytotoxic one. The non-cytotoxicity of COL/TA LbL films was confirmed on a human gingival fibroblast primary cell line for up to 9 days [30]. These results validate the choice of TA and COL as components of LbL films for biomaterial coatings.

## 3. Conclusions

In this study, COL-based films were self-assembled with TA through H-bonds, PSS, and HEP through electrostatic interactions by the LbL method and optimized by changing the pH value, the dipping time, and NaCl concentration of the solutions. COL/TA films fabricated with COL solutions at pH 5 and TA solutions at pH 4.4 showed better reproducibility and allowed the surface to be covered after three pairs of layers. COL/PSS LbL films were built at pH 5 and COL/HEP films at pH 3 and 50 mM NaCl covering the whole surface with four and seven pairs of layers, respectively. In contact with a physiological medium, a loss of 30% in the thickness of COL/TA led to a release of TA up to 1 µM in the supernatant. No loss in thickness was observed in the case of COL/PSS films, and 60% was observed for COL/HEP films. Surfaces fully covered by the films allowed us to evaluate their cytotoxicity towards human fibroblasts seeded for 22 h. COL/TA films showed no cytotoxicity in comparison with COL/PSS and COL/HEP.

## 4. Materials and Methods

### 4.1. Materials

HEP sodium salt from porcine intestinal mucosa (≥180 USP units/mg, BioReagent, suitable for cell culture), PSS (Mw ~ 70,000 g/mol), sodium citrate tribasic dehydrate (ACS reagent), citric acid monohydrate (ACS reagent), 4-Nitrophenyl phosphate disodium salt hexahydrate (*p*NPP), sodium acetate anhydrous, and Trizma^®^ base were purchased from Sigma-Aldrich, St. Louis, MO, USA. Eagle’s Minimum Essential Medium (EMEM, ATCC^®^ 30-2003™) and BJ fibroblast cell (ATCC^®^ CRL-2522™) were purchased from ATCC, Manassas, VA, USA. Dulbecco’s phosphate-buffered saline (PBS, L0615) and Trypsin-EDTA 1X in PBS (L0940) were purchased from Dominique Dutscher, Bernolsheim, France. TA was obtained from Alfa Aesar, Haverhill, MA, USA. Purified type 1 COL was purchased from Symatese, Chaponost, France. Sodium dodecyl sulfate (SDS) was obtained from Prolabo, Warszawa, Poland. Sodium chloride was obtained from VWR, Radnor, PA, USA. Triton^®^ X-100 was purchased from Fisher Scientific, Waltham, MA, USA. Milli-Q water (18.2 MΩ·cm at 25 °C) supplied by Advantage A10 (MERCK) was used in the entire study. All the materials were used as received.

### 4.2. Preperation of Buffers and Polyelectrolyte Solutions

Different pH values of 100 mL of citrate buffer used in the study were prepared by mixing *x* mL of 0.1 M sodium citrate tribasic dehydrate solution and *y* mL of 0.1 M citric acid monohydrate solution. pH 3 (*x* = 18; *y* = 82), pH 3.8 (*x* = 36.5; *y* = 63.5), pH 4 (*x* = 41; *y* = 59), pH 4.4 (*x* = 50.5; *y* = 49.5), pH 5 (*x* = 65; *y* = 35), and pH 5.4 (*x* = 74.5; *y* = 25.5) were prepared. Physiological medium was prepared by adjusting the pH value to pH 7.4 through adding 1 M HCl into 150 mM NaCl 10 mM tris buffer. For the cytotoxicity tests, all the buffers used for film buildup were filtered through a 0.2 µm filter (Sarstedt, no. 831826001). All polyelectrolyte solutions were prepared freshly prior to use, except the COL solution, which was prepared by dissolving the COL in citrate buffer overnight and by filtering the obtained solution through a 0.45 µm filter (Sarstedt, no. 831826) to remove any remaining impurities.

### 4.3. Assembly of Multilayers

#### 4.3.1. Substrate Preparation

Multilayer films were constructed through the consecutive deposition of COL and a partner (TA, PSS, and HEP) on silicon wafers (wafernet, inc) or 12 mm diameter glass slides. Silicon wafers were sonicated in ethanol/water (50/50 *v*/*v*) solution in an Elmasonic S ultrasonic cleaning machine (Elma) for 10 min at room temperature. For glass substrates, a cleaning procedure consisting of dipping in a 0.01 M SDS solution for 15 min at 70 °C, rinsing extensively with Milli-Q water, dipping in a 0.1 M HCl solution for 15 min at 70 °C, and rinsing extensively with Milli-Q water was applied. Both substrates, the silicon wafers and the glass coverslips, were activated by plasma cleaning (Harrick) for 3 min under vacuum.

#### 4.3.2. Deposition of Multilayer Films

The plasma-activated substrates were first dipped in a 0.2 mg/mL COL citrate buffer solution. Then, the COL-coated substrates were dipped in a 0.5 mg/mL TA, 0.2 mg/mL PSS, or 0.2 mg/mL heparin citrate buffer solution. This procedure was repeated to construct (COL/TA)*_n_*, (COL/PSS)*_n_*, and (COL/HEP)*_n_* multilayered films, where *n* is the number of pair of layers. The dipping time of the deposition step, pH value, and NaCl concentration of the solution were varied.

In the case of non-dried film, the substrates were rinsed after each deposition step with the same buffer used as the previous adsorbed polyelectrolyte solution. In the case of dried films, the substrates were rinsed between each deposition step by dipping for 2 min in HCl-adjusted water with the same pH as the previous adsorbed polyelectrolyte solution and dried after each rinsing step with compressed air. Similar building steps were applied in the case of glass slides. The substrates were further placed in 24-well plates (Greiner bio-one, 662160). The deposition step was carried out by immersing the substrate in 300 µL of polyelectrolyte solution for 20 min. Between each deposition step, the polyelectrolyte solution was removed, and the film was rinsed with 1 mL of citrate buffer (the same buffer as the final adsorbed polyelectrolyte solution) for 2 min. TA-based films were covered with aluminum paper to avoid any photooxidation of TA. All the films mentioned above were built at room temperature.

#### 4.3.3. Film Thickness by Ellipsometry

The film thickness was measured after a drying step by a Plas-Mos Ellipsometer (SD2300, Plasmos) with an incident laser beam (632.8 nm) and a constant incidence angle of 45°. The refractive index was assumed to be constant, *n* = 1.465, and was set during the measurement. The thickness values reported represent the mean and standard deviation of the measurement conducted on three different samples, with each measurement being the average of 10 values from 10 different areas of the sample.

### 4.4. AFM Imaging of Multilayer Film

The topography and cross-section profile of the multilayer films were observed by an AFM Bioscope Catalyst (Bruker, Billerica, MA, USA) under room temperature. MLCT cantilevers (made with non-conductive silicon nitride, Brucker) with a spring constant of 0.1 or 0.3 N/m were used. The AFM was operated in contact mode in either a dry state or a liquid state (the same buffer as the final adsorbed polyelectrolyte solution) for each sample. The AFM image was obtained at a scan rate of 2 Hz.

### 4.5. Stability Test in Physiological Medium

All stability tests were conducted on three different samples. To this aim, the films deposited on silicon wafers were dipped in 10 mM tris–150 mM NaCl pH 7.4 buffer for a fixed dipping period at room temperature, followed by a rinsing step in water at pH 7.2. The film thickness was measured by ellipsometry after a drying step. The loss of film thickness was calculated using Equation (1).

(1) $$Loss\;in\;thickness\;\left(\%\right)=\frac{Initial\;thickness-final\;thickness}{Initial\;thickness}\times 100$$

### 4.6. TA Release in Physiological Solutions

COL/TA films deposited on 12 mm diameter glass slides were used to follow the TA release during their immersion in 10 mM tris–150 mM NaCl pH 7.4 buffer for a fixed incubation period under room temperature. The absorbance of the supernatant was measured at 277 nm in UV-Star^®^ 96-well plates (Greiner bio-one) with a spectrofluorometer (SAFAS Xenius XC). The COL used in the study showed no absorption at this wavelength. First, a calibration curve of TA was obtained by measuring the absorbance intensity at 277 nm for different concentrations of TA in the physiological solution. The films were covered with aluminum paper during the test. All tests were conducted on three different samples. The TA concentration was calculated using standard calibration.

### 4.7. In Vitro Cytotoxicity Assay

The cytotoxicity of the films, i.e., (COL/TA)_3_, (COL/TA)_3_-COL, (COL/PSS)_4_, (COL/PSS)_4_-COL, (COL/HEP)_6_-COL, and (COL/HEP)_7_ film, was assessed via an acidic phosphatase assay on human foreskin fibroblast cells (BJ) ATCC^®^ CRL-2522™. The experiments were conducted in triplicate. Each tested film was built on a 12 mm diameter glass slide in a 24-well plate (Greiner bio-one, 662160) the day before the assay. After a rinsing step with 1 mL of PBS, 50,000 cells were seeded in each well and incubated onto film at 37 °C with 5% CO_2_. The cells were cultivated in EMEM supplemented with 10% fetal bovine serum (FBS) and 1% penicillin/streptomycin. After an incubation time of 22 h, the films were washed with 1 mL of PBS. Then, 300 µL of 100 mM sodium acetate (NaOAC) solution with 1% Triton X-100 and 0.5 mg/mL of *p*NPP were added and incubated at 37 °C with 5% CO_2_ for 1 h. Then, 5 µL of 1 M NaOH was added to stop the reaction between the phosphatases and *p*NPP and to deprotonate the *p*-nitrophenol, the product of the reaction, to give yellow-colored *p*-Nitrophenolate. To evaluate the amount of *p*-Nitrophenolate, 100 μL of the reaction media in each well was transferred to a 96-well plate. The absorbance at 405 nm was recorded with a spectrofluorometer used in the absorbance mode (SAFAS Xenius XC). Cells seeded on TCPS were used as a control. The absorbance of each film was then subtracted with the absorbance of the TCPS (tissue culture polystyrene plate) which was not cell seeding. For each sample, the test was run without cells to check that the film did not hydrolyze *p*NPP by itself.

## Figures and Tables

**Table 1:** Dry and wet films’ thickness of (COL/TA)_3_ and (COL/TA)_3_-COL measured by ellipsometry and AFM, respectively.

Films	Dry Thickness by Ellipsometry (nm) ^a^	Wet Thickness by AFM (nm)
Dried (COL/TA)_3_	48 ± 6	57 ± 27 ^b^
Non-dried (COL/TA)_3_	17 ± 2	34 ± 15 ^c^
Dried (COL/TA)_3_-COL	49 ± 5	60 ± 16 ^b^
Non-dried (COL/TA)_3_-COL	20 ± 7	23 ± 7 ^c^

^a^ Average value of at least three different samples. ^b^ Average value over two independent areas (thick and thin part) in one sample. ^c^ Average value over one area in one sample.

## Data Availability

Data are contained within the article or Supplementary Material.

## Supplementary Materials

The following supporting information can be downloaded at: https://www.mdpi.com/article/10.3390/gels9030192/s1, Figure S1: Evolution of the thickness of (a) (COL/PSS)_7_ and (b) (COL/HEP)_7_ films measured by ellipsometry in triplicate in the dry state built at (1) different pH and (2) different NaCl concentrations for (COL/PSS) at pH 5 and (COL/HEP) at pH 3 with 20 min of dipping; Figure S2: (a) (COL/PSS)_4_ and (b) (COL/HEP)_7_ films imaged by AFM in contact mode and dry state with their (1) surface topography, (2) AFM 3D images and (3) cross-section profile after scratching; Figure S3, Thickness loss of dried (a) (COL/HEP)_7_ and (b) (COL/PSS)_7_ films in physiological solution measured by ellipsometry in a dry state; Table S1, Film thicknesses of (COL/PSS)_4_ and (COL/HEP)_7_ measured by AFM and ellipsometry.
