# Supplementary material for: Physical Chemistry Study of Collagen-Based Multilayer Films
Source: Gels. 2023 Mar 2;9(3):192. doi: 10.3390/gels9030192 (PMC10048292; doi:10.3390/gels9030192)
Supplement: Supplementary file 1 [file gels-09-00192-s001.zip › gels-2208678-supplementary.pdf]

gels

Supplementary Materials

Physical Chemistry Study of Collagen-Based Multilayer Films

Yi-Wei Chen [1], Muhammad Haseeb Iqbal [1], Florent Meyer [2,3], Vincent Ball [2,3] and Fouzia Boulmedais [1,*]

[1] Institut Charles Sadron (UPR 22), Université de Strasbourg, CNRS, 67034 Strasbourg, France
[2] Centre de Recherche en Biomédecine de Strasbourg, Institut National de la Santé et de la Recherche Médicale, 67085 Strasbourg, France
[3] Université de Strasbourg, Faculté de Chirurgie Dentaire, 67000 Strasbourg, France
* Correspondence: fouzia.boulmedais@ics-cnrs.unistra.fr

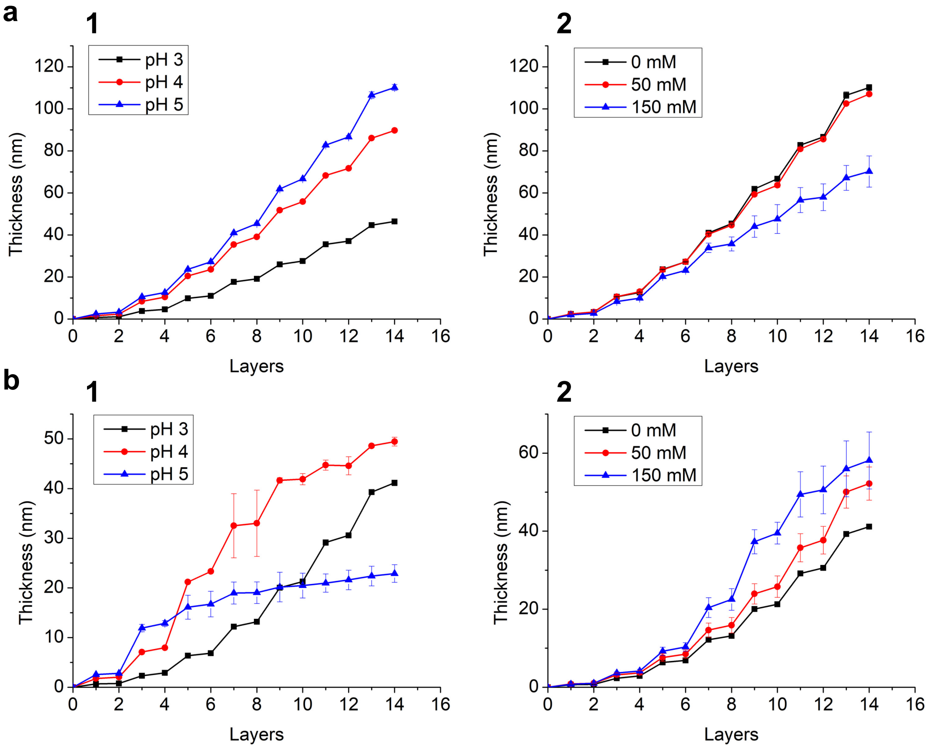

Figure S1: Buildup of COL/PSS films and COL/HEP films. Evolution of (a) $(COL/PSS)_7$ and (b) $(COL/HEP)_7$ films thickness measured by ellipsometry in triplicate in dry state built at (1) different pH and (2) different NaCl concentrations for $(COL/PSS)_7$ at pH 5 and $(COL/HEP)_7$ at pH 3 respectively with 20 min of dipping.

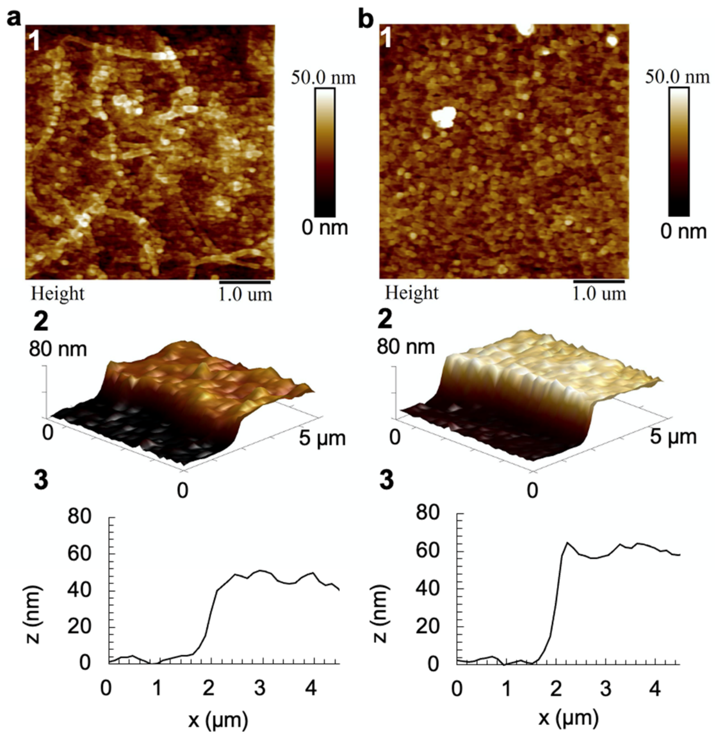

Figure S2: (a) (COL/PSS)4 and (b) (COL/HEP)7 films imaged by AFM in contact mode and dry state with their (1) surface topography, (2) AFM 3D images and (3) cross-section profile of scratched film in their respective pH buffer.

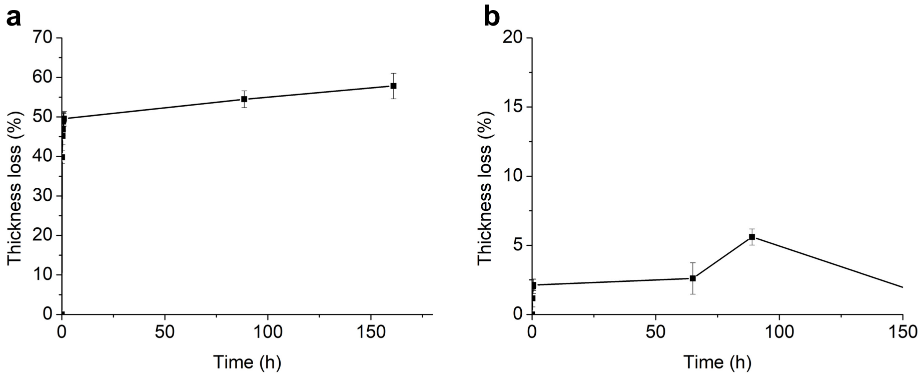

Figure S3: Thickness loss of dried (a) (COL/HEP)7 and (b) (COL/PSS)7 films on silicon in physiological solution measured by ellipsometry in the dry state.

Table S1: Films thickness of (COL/PSS)4 and (COL/HEP)7 measured by AFM and ellipsometry.

Films	Thickness by ellipsometry (nm)[a]	Thickness by AFM (nm)[b]
Dried (COL/PSS)4	45.4 ± 0.3	47.3 ± 5.5
Dried (COL/HEP)7	52.2 ± 4.2	66.9 ± 2.3

[a]average value of at least three different samples. [b]average value over one area in one sample.
